# Prevalence and Distribution of Musculoskeletal Symptoms in Cystic Fibrosis and Impacts on Daily Life

**DOI:** 10.1002/ppul.27418

**Published:** 2024-12-02

**Authors:** Elizabeth Clarke, Julia Taylor, Pippa Watson, Jane Freeston, Andrew Jones, Daniel Peckham, Alex Horsley

**Affiliations:** ^1^ Division of Infection, Immunity & Respiratory Medicine, Faculty of Biology, Medicine and Health University of Manchester Manchester UK; ^2^ Rheumatology Department Royal Alexandra Hospital, NHS Greater Glasgow and Clyde; ^3^ Manchester Adult Cystic Fibrosis Centre Wythenshawe Hospital, Manchester Foundation NHS Trust Manchester UK; ^4^ Rheumatology Department Wythenshawe Hospital, Manchester Foundation NHS Trust Manchester UK; ^5^ NIHR Leeds Biomedical Research Centre and Department of Rheumatology Leeds Teaching Hospitals NHS Trust Leeds UK; ^6^ Leeds Institute of Rheumatic and Musculoskeletal Medicine University of Leeds Leeds UK; ^7^ Leeds Adult Cystic Fibrosis Unit Leeds Teaching Hospitals NHS Trust Leeds UK

**Keywords:** cystic fibrosis, musculoskeletal pain

## Abstract

**Background:**

Musculoskeletal problems are reported in the literature as a common problem for people with cystic fibrosis, with a range of aetiologies including an inflammatory arthritis. However, accurate data on the presentations and prevalence are lacking. The aim of this cohort study was to describe the scale and impact of musculoskeletal symptoms in CF.

**Methods:**

A collaboratively designed questionnaire was administered to adults attending two large UK CF centres. Data collected evaluated scale and impact of musculoskeletal symptoms.

**Results:**

Results were obtained from 489 patients (response rate 59%). Of these, 49% reported that musculoskeletal symptoms impacted their activities of daily living in the previous year. Back pain was common, occurring in 44% of participants in the preceding week. The knee was the most commonly affected painful peripheral joint, with 26% of participants reporting knee pain within the last week rising to 50% within the last year. Early morning stiffness and joint swelling were markedly less common, suggesting that the majority of musculoskeletal pain in CF is not due to an inflammatory arthritis but is due to other factors.

**Conclusion:**

Musculoskeletal problems are common in CF and frequently affect activities of daily living. Symptoms of inflammatory arthritis occurred in only a small minority of individuals. A focused approach to characterising and clarifying the aetiology of musculoskeletal symptoms is needed to inform the management of these disabling symptoms.

## 1 Introduction

Musculoskeletal (MSK) pain is common in cystic fibrosis (CF) and can be both disabling and burdensome [1, 2]. Existing literature suggests that MSK pain may be under‐reported by those living with it, and also that pain more generally in CF correlates with mortality outcomes [1, 2, 3, 4]. The aetiology of MSK pain in people with CF is often multifactorial and reflects both CF‐related disease as well conditions commonly seen in the non‐CF population [5]. This spectrum of causes includes inflammatory arthritis, biomechanical issues associated with muscle function, osteomalacia, and musculoskeletal issues seen more commonly in diabetes such as frozen shoulder [6, 7, 8]. CFTR dysfunction has both direct (via its expression in muscle) and indirect impacts on muscle function. Factors such as inflammation, nutrition (low body mass is associated with low muscle mass), physical activity levels, and pharmacological elements such as the proximal myopathy caused by use of systemic corticosteroids, all play important contributing roles in muscle function [7].

Inflammatory arthritis is characterised by inflammation of the synovial lining of the joint, with swelling and increased blood flow to the area. An inflammatory arthritis was first reported in CF in 1978, but this appears to be much less common than the reported incidence of MSK symptoms [8, 9, 10]. The presence of an inflammatory arthritis requires early rheumatological assessment to enable appropriate management to minimise disease progression and improve rates of remission [11]. Equally important however is correct identification and treatment of noninflammatory, biomechanical and other presentations to reduce impact on quality of life as well as to enable full participation in other CF treatments including airway clearance and exercise [12]. Quantifying the prevalence and impact of musculoskeletal pain in people with CF is an essential first step to better assessment and management of these issues.

## 2 Methods

### 2.1 Study Design and Population

A questionnaire was designed to evaluate the extent of musculoskeletal symptoms in the CF population. This was administered in two large adult CF centres in the UK comprising 830 patients between the end of May 2017 and the start of November 2017 (5 months), before the advent of CFTR modulator triple therapy, and before dual therapy combinations were widely available in the UK. All patients aged over 16 years with a diagnosis of CF attending either of the adult CF centres were eligible for inclusion, and the questionnaire was offered by clinic staff at outpatient clinic appointments. Additional data were obtained from medical records including genotype, FEV_1_% (best in last 6 months), colonising organisms, previous rheumatological diagnoses, treatment with disease modifying antirheumatic drugs, and recent (last 6 months) or current systemic corticosteroid use. Ethical approval was granted by the North West–Greater Manchester Research Ethics Committee (REC number 17/NW/0295).

### 2.2 Development of the Questionnaire

**Figure 1:**
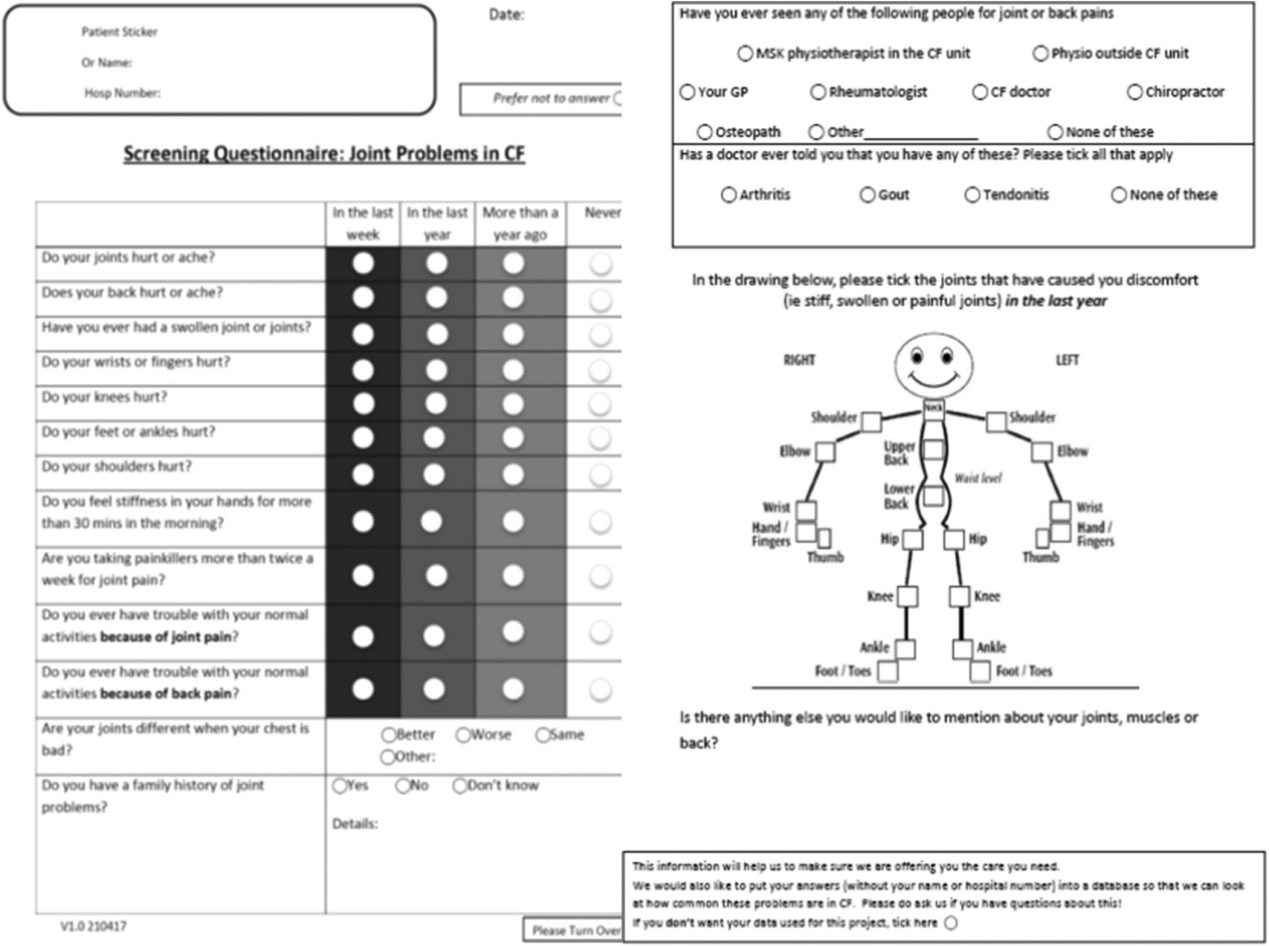
The questionnaire developed for recording musculoskeletal symptoms in adults with cystic fibrosis.

The questionnaire was custom designed by combining elements from previously validated screening questionnaires for patients with skin psoriasis (at risk of psoriatic arthritis) with the views of a range of experienced health professionals who care for people with CF and MSK problems. This included specialist physiotherapists and senior physicians specialising in Rheumatology, Orthopaedics and CF. This was further modified following input from four people with CF, including two with MSK symptoms. Elements from both the Early Arthritis for Psoriatic patients (EARP) and the Psoriasis Epidemiology Screening Tool (PEST) were used [13, 14]. Permission to use the PEST tool mannequin was granted. The final questionnaire (Figure 1) consisted of 12 questions regarding symptoms, one question on relevant family history, one question regarding help‐seeking with regard to MSK symptoms, one question regarding previous MSK diagnoses, and the mannequin from the PEST questionnaire. A free‐form space for participant comments was also provided. Consent for inclusion of data from the questionnaire into a study was ascertained by means on an opt‐out tick box option, which was felt appropriate for the low risk nature of this study.

### 2.3 Data Analysis

Data were collated using the Castor database system (Castor Electronic Data Capture https://castoredc.com) and analysed using Stata (StataCorp. 2017. *Stata Statistical Software: Release 15*. College Station, TX: StataCorp LLC.), Microsoft Excel, and Graphpad (GraphPad Prism version 8.0.0 for Windows, GraphPad Software, San Diego, California USA, www.graphpad.com). Demographic data were expressed as percentages of the group, or median and interquartile range. Most of the questionnaire data were discrete data. Correlations were assessed using the Pearson Chi^2^ test, significance level was set at *p* < 0.05.

## 3 Results

The questionnaire was completed by 490 people, one participant opted out of data being used for research. Results are therefore available for the outcomes of symptom burden for 489 patients representing 59% of the total eligible CF population of the two participating CF centres. Summary demographic and clinical data are shown in Table 1. The population was largely reflective of that regularly attending the participating clinics, with a median age of 29 years, median FEV_1_ of 59% predicted, the majority (54%) being homozygous for the Phe508del CFTR mutation, and 69% being chronically infected with Pseudomonas aeruginosa. 57% of respondents were male. Rates of long‐term systemic corticosteroid use were 7%, with 13% of patients having received oral corticosteroids in the previous month.

**Table 1:** Demographic and CF data.

Demographic and clinical data	
Age (years) Median (IQR)	29 (23–37)
Gender male *N* (%)	277 (57)
Genotype: homozygous Phe508del *N* (%)	262 (54)
FEV_1_ % predicted (best in last 6 months) Median (IQR)	59 (41–77)
Exocrine pancreatic insufficiency *N* (%)	421 (86)
Diabetes mellitus *N* (%)	171 (35)
Systemic corticosteroids prescribed in the last month, including long term prescriptions *N* (%)	68 (13)
Long term systemic corticosteroids (> 1 month) *N* (%)	34 (7)
Pseudomonas aeruginosa infection *N* (%)	335 (69)

**Figure 2:**
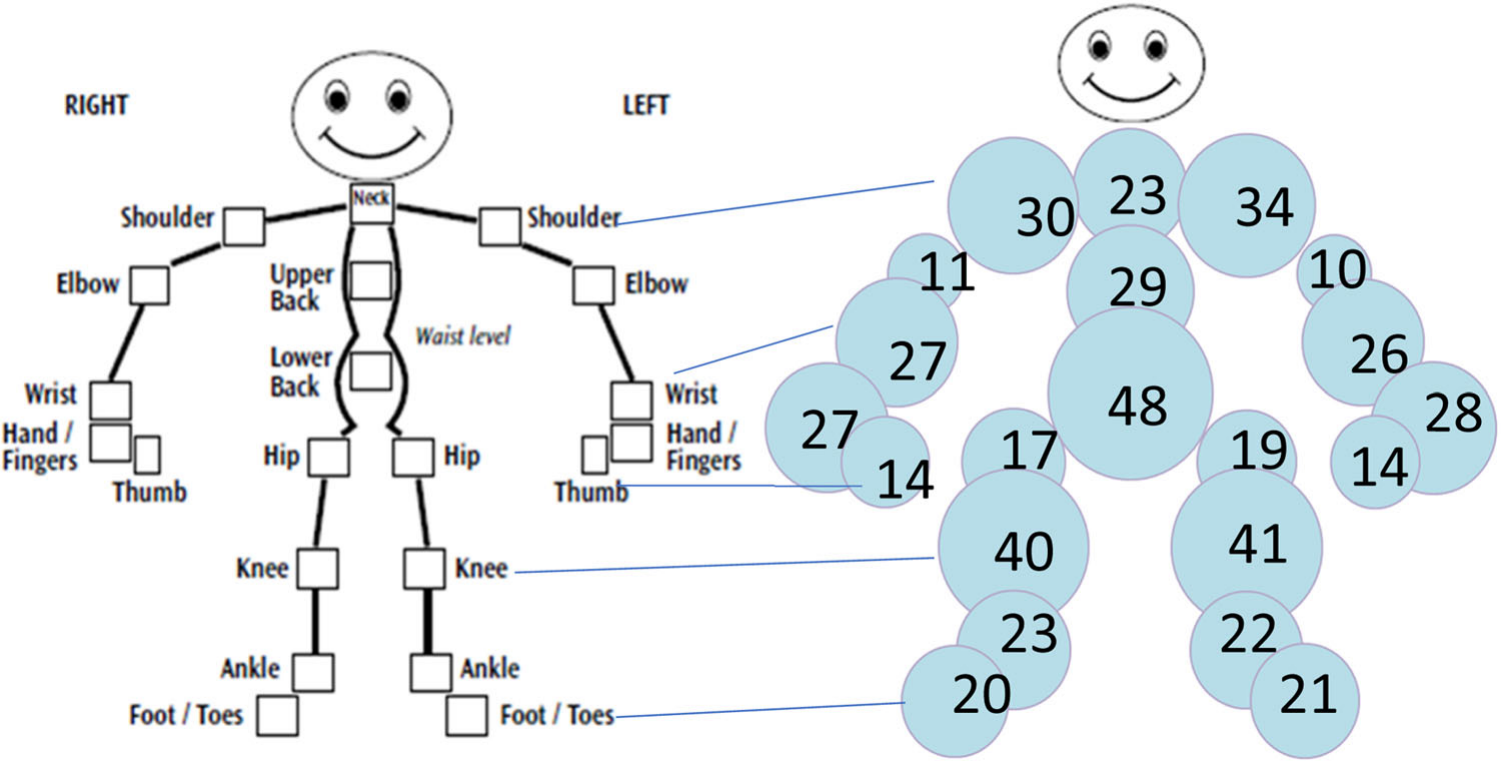
Mannequin data showing the frequency of different joint symptoms in adults with cystic fibrosis. The size of the bubble over each joint is proportionate with the number of respondents identifying that joint as symptomatic. Numbers in each bubble represent the percent of patients who identified each joint as being stiff, swollen or sore in the last year.

Question responses are shown in Table 2. Pain in back or joints in the last week was reported by 274 (56%) participants, and cumulatively by 377 (77%) in the last year. 132 (27%) reported a swollen joint in the last year. Mannequin data were available for 476 out of the 489 participants (Figure 2). Participants identified a median of four joints (IQR 1–8) that had caused discomfort in the last year. 79 (17%) participants ticked the box to identify that they had no joint discomfort (added to ensure that an accidentally unfilled mannequin was not counted as “no joint pain”). Internal validity was assessed by comparing written question data with mannequin data for the 476 participants who completed both sides of the questionnaire. This showed a difference of between 3%–6% in answers. Both joint and back pain were more likely to be reported as present in the written question than on the mannequin.

**Table 2:** Overview of question responses.

Question	In the last week *n* (%)	In the last year *n* (%)	More than a year ago *n* (%)	Never *n* (%)
Do your joints hurt or ache?	198 (40)	116 (24)	23 (5)	150 (31)
Does your back hurt or ache?	213 (44)	113 (23)	31 (6)	127 (26)
Have you ever had a swollen joint or joints?	56 (11)	78 (16)	57 (12)	291 (60)
Do your wrists or fingers hurt?	117 (24)	110 (22)	23 (5)	236 (48)
Do your knees hurt?	128 (26)	116 (24)	36 (7)	208 (43)
Do your feet or ankles hurt?	92 (19)	94 (19)	43 (9)	254 (52))
Do your shoulders hurt?	111 (23)	93 (19)	41 (8)	239 (49)
Do you feel stiffness in your hands for more than 30 min in the morning?	49 (10)	32 (6)	20 (4)	382 (78)
Are you taking painkillers more than twice a week for joint pain?	87 (18)	57 (12)	25 (5)	320 (65)
Do you ever have trouble with your normal activities because of joint pain?	85 (17)	93 (19)	178 (36)	270 (55)
Do you ever have trouble with your normal activities because of back pain?	83 (17)	105 (21)	188 (38)	257 (52)

With regard to associations between respiratory and MSK symptoms: 308 (63%) of participants reported no difference in joint symptoms with respiratory exacerbations of their CF; one reported joint symptoms to be better during exacerbations; and 114 (23%) reported worsening of joint symptoms. In addition to this, 56 (11%) gave the answer “other,” with several comments suggesting that this was variable in their experience.

No correlations with joint symptoms or impact on activities of daily living was found with lung function, microbiological status, or CF related diabetes. Female gender was associated with higher levels of MSK symptoms, with more frequent joint and back pain (combined symptoms in last 12 months: 84% in females versus 72% males, *p* = 0.002), and more impact on activities of daily living (58% vs. 42% *p* < 0.001). In addition to this, female gender was also more likely to be associated with symptoms associated with inflammatory arthritis as opposed to mechanical joint pain: 15% of women and 8% of men reported the combination of both swollen joints and early morning stiffness, *p* = 0.018. Corticosteroid prescription correlated with a small increase in joint pain in the last week, and more joints identified on the mannequin (average 6.7 joints vs. average of 5 for those not on corticosteroids).

The following diagnoses were recorded in participant case notes: CF‐associated arthritis 48 patients (10%); gout 33 (7%); tendinitis 9 (2%); osteoarthritis 3 (0.6%); rheumatoid arthritis 1 (0.2%). 29 patients (6%) had other diagnoses recorded for their joint symptoms. No patients had a diagnosis of ankylosing spondylitis or psoriatic arthritis. A total of 277 (56%) patients reported seeking help from at least one source, most commonly from a physiotherapist (124 patients, 26%). 85 (17%) patients reported seeking help from a CF physician. Activities of daily living were impacted by joint or back pain in 49% within the last year.

**Figure 3:**
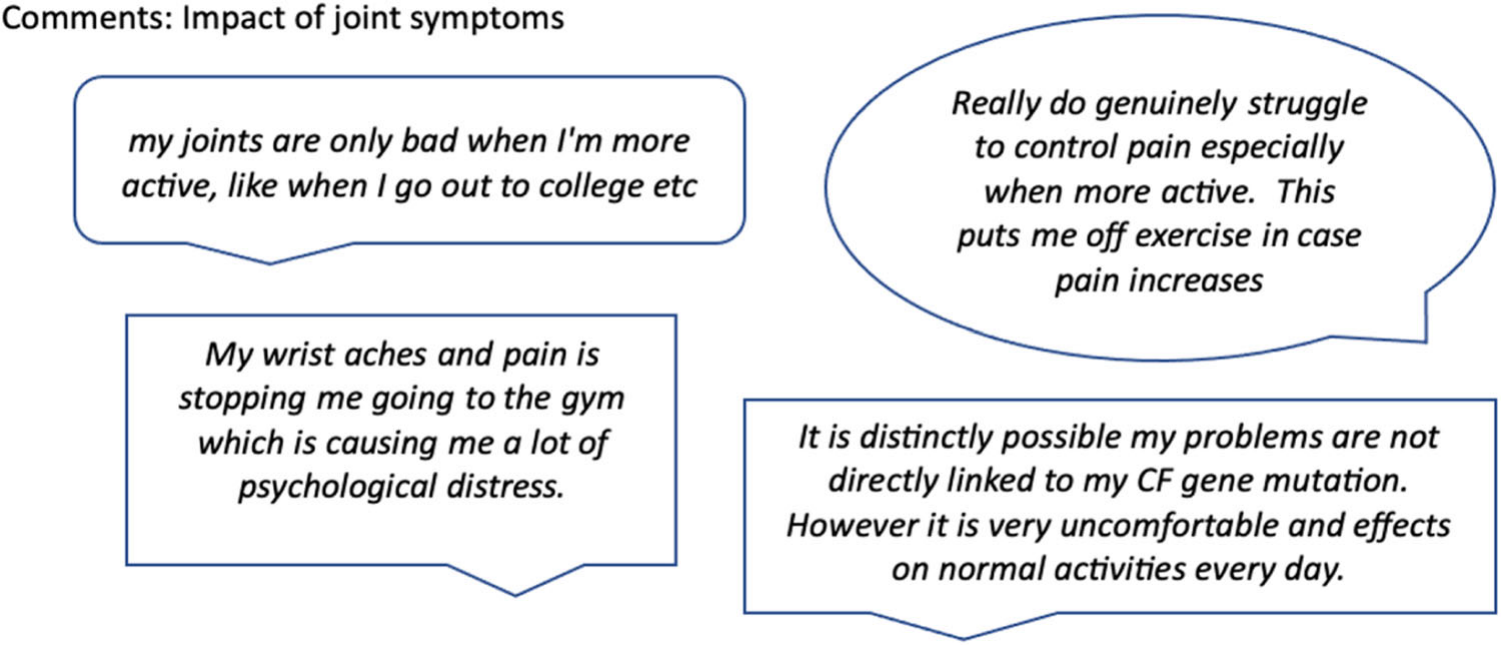
Participant comments on the theme of impact of joint symptoms.

A total of 133 participants offered comments in the free‐form space. A proportion related symptoms to specific injuries or events, others comment on how difficult things are when their joints are bad. Data collected was analysed thematically following guidelines created by Braun and Clarke [13]. Themes identified were: inflammatory symptoms, noninflammatory symptoms, impact of symptoms, help‐seeking, and associations with chest disease. Some impact of symptoms comments are shown in Figure 3.

## 4 Discussion

Musculoskeletal pain is an extremely common complaint in this group and has a significant impact on the lives and wellbeing of many people living with CF. The patient questionnaire was not designed to determine the aetiology of MSK symptoms but our experience suggests that much of this is likely to be mechanical, for which there are a number of possible mechanisms in CF including the expression of CFTR in muscle, the use of systemic corticosteroids, the impact of nutritional status and diabetes [7, 15].

The knee was the most common peripheral joint identified as being painful within the last week (26% of participants) and in the last year (50%). Wrist and finger pain was marginally less common than knee pain but 48% reported pain in the last year, and 24% reported this in the last week. Associated mannequin data showed one or both wrist joints marked by 29%, and one or both hands in 30%. At least one area of hand or wrist was marked on the mannequin by 40% of participants. Shoulder pain was reported by 204 (42%) in the last year, and in CF may have different incidence than in the non‐CF population for a variety of reasons including postural changes associated with coughing, and changes seen in shoulder joints in diabetes such as a propensity to develop adhesive capsulitis (frozen shoulder) [6]. Very few marked these symptoms as being problematic more than a year ago—either they have caused problems in the last year or not at all. This may represent a chronicity of musculoskeletal pain where it is present.

It is reassuring that only a small proportion of participants reported joint swelling or early morning stiffness for more than 30 min. These are both more closely linked with inflammatory joint disease than the other questions and supports the hypothesis that inflammatory joint disease is not the most frequent cause of musculoskeletal pain in people with CF. It highlights the need for detailed assessment and consideration of differential diagnosis in all patients presenting with MSK pain.

Whilst there is considerable heterogeneity of causes of MSK pain represented here, there was not a strong association shown between MSK symptoms and exacerbations of chest disease or between lung function and MSK symptom severity. MSK pain does not primarily affect people with CF with end stage lung disease but is common throughout the cohort.

The MSK diagnoses identified in the medical case notes included 10% with CF related arthropathy. This is a poorly defined condition and some of these patients may in fact have had other diagnoses causing or contributing to their symptoms. On the other hand, some of those with other diagnoses listed—notably the palindromic arthropathy, suspected inflammatory arthritis and seronegative arthritis cases—may in fact have a CF associated arthritis. True prevalence of CF related arthropathy is not possible to determine from these data alone. Notable is one diagnosis not recorded: two participants had Crohn's disease for which they were on azathioprine. Both report some joint symptoms, one reported an improvement on commencing azathioprine in the comments, the other reported that her joints were worse when her Crohn's flared. Neither had a diagnosis of inflammatory bowel disease related arthritis recorded.

The corticosteroid prescriptions are notable in the setting of this research question due to implications for proximal muscle strength, sleep quality with its impact on pain, and on the difficulties of managing diabetes which contributes to disrupted sleep as well as to pathologies such as adhesive capsulitis. Whilst corticosteroid therapy can be used to treat inflammatory disease, inflammatory symptoms constitute a minority of those reported here. Indications for corticosteroid prescription were not recorded.

Activities of daily living were reported to be impacted within the last year by 49% of respondents. This has a wide variety of potential impacts, including loss of independence, concern about potential loss of independence in relapsing and remitting cases, difficulties carrying out societal obligations, and difficulty completing treatments. Opening blister packs, putting together a nebuliser, and doing exercise are all more difficult with painful joints, and this was reflected in participant comments. This contrasts with the number of participants who reported taking analgesia, which is notably lower than those reporting impact on activities of daily living. Some comments suggest reasons for this—concerns regarding interactions between analgesia and other health issues, as well as a belief that analgesia should be “last resort” rather than used to enable normal activity or better sleep. Underplaying symptoms and acceptance of symptoms as part of life may also play roles. For example, the comment “they are worse when I am more active” is more concerning given that it is qualified by identifying “active” as attending college (Figure 3).

This impact on exercise, with respondents identifying MSK pain as a barrier, is relevant for longer term outcomes, especially given the role of exercise in maintaining respiratory health, but this will also impact extra‐pulmonary features such as osteoporosis and diabetes.

Population‐based studies of musculoskeletal pain often report pain increasing with age; the age group best represented here tending to have the least. A study that reported chronic musculoskeletal pain in 31.4% of the general adult population aged 20–74 years noted a significant trend of pain increasing with age [16]. The levels reported here by adults with CF are difficult to compare due to the difference in ages best represented but seem likely higher. Cimmino et al note the difficulty in comparing studies looking at rates of musculoskeletal pain, as well as identifying the need going forwards for the impact on daily life to be considered [17].

Life for many people living with CF has changed significantly since this survey was completed with the introduction of highly effective CFTR modulator therapy. There is a case report of improvement with highly effective modulators in a child with CFA, which is encouraging [18]. Registry data often groups musculoskeletal symptoms with metabolic bone disease, making it difficult to interpret with regard to the musculoskeletal symptoms [19]. These findings remain relevant however for a number of reasons. Firstly, CFTR modulators are not available to all, especially when CF is considered globally. Secondly, the impact of years lived with CF without modulator therapy may have long lasting effects even where some impacts are reversed, and we do not yet understand enough about the spectrum of MSK disease in CF to assess this accurately. Thirdly, the improvements in life expectancy are such that we may expect to see an increase in diseases associated with ageing in the CF population, and these include changes in incidence of inflammatory arthritis as well as osteoarthritis and sarcopenia. The need to support people living with CF and MSK symptoms is likely to therefore increase over time. Having good quality data on the size and nature of the problem is an essential first step to better diagnosis and management. Limitations of this study include its design as a retrospective questionnaire, relying on participant recall of symptoms, its distribution to those attending adult clinic only, and a lack of full clinical assessment alongside the results. There was however good coverage of both centres, with over half of all patients participating and resulting in the largest data set of patient‐reported MSK symptoms in CF.

## 5 Conclusions

Musculoskeletal symptoms are common in people with CF and have a significant propensity to disrupt activities of daily living. These patient‐reported symptoms highlight a significant area of unmet need both in terms of clinical research and in terms of addressing the presence of musculoskeletal symptoms within a clinical encounter. Those with symptoms suggesting an inflammatory arthritis form a minority, such that research focused purely on CF associated arthritis will not serve a large number of those living with CF and musculoskeletal pain. Addressing the spectrum of MSK disease in CF requires engagement with multidisciplinary teams to create multimodal tailored treatment plans and is vital to reduce the impact of MSK symptoms on daily life for many people living with CF.

## Author Contributions

**Elizabeth Clarke:** conceptualization, methodology, software, data curation, investigation, validation, formal analysis, project administration, writing–original draft, writing–review and editing. **Julia Taylor:** conceptualization, writing–review and editing, methodology. **Pippa Watson:** conceptualization, methodology, supervision, writing–review and editing. **Jane Freeston:** supervision, writing–review and editing, conceptualization, methodology. **Andrew Jones:** conceptualization, supervision, writing–review and editing, funding acquisition. **Daniel Peckham:** conceptualization, methodology, funding acquisition, writing–review and editing. **Alex Horsley:** conceptualization, methodology, writing–review and editing, supervision, funding acquisition.

## Conflicts of Interest

The authors declare no conflicts of interest.

## Data Availability

The data that support the findings of this study are available from the corresponding author upon reasonable request.
